# Beyond Broca and Wernicke: Epilepsy surgery in the language areas

**DOI:** 10.1002/epd2.70282

**Published:** 2026-05-30

**Authors:** Carmen Barba, Alessandro De Benedictis, Veronica Pelliccia, Domenico Tortora, Federico Melani, Alessandro Consales, Domenica Immacolata Battaglia, Elisabetta Cesaroni, Lorenzo Ferri, Alice Noris, Matteo Pugnaghi, Michele Rizzi, Giuseppe Bertini, Flavio Villani, Laura Tassi, Commission of Epilepsy Surgery, Italian League against Epilepsy

**Affiliations:** ^1^ Department of Neuroscience, Psychology, Drug Research, and Child Health (NEUROFARBA) University of Florence Florence Italy; ^2^ Neuroscience and Human Genetics Department Meyer Children's Hospital IRCCS, Full Member of the European Reference Network EpiCARE Florence Italy; ^3^ Neurosurgery Unit Bambino Gesù Children's Hospital IRCCS Rome Italy; ^4^ “Claudio Munari” Epilepsy Surgery Centre ASST Niguarda Hospital Milan Italy; ^5^ Neuroradiology Unit IRCCS Istituto Giannina Gaslini Genoa Italy; ^6^ Division of Neurosurgery IRCCS Istituto Giannina Gaslini Genoa Italy; ^7^ Department of Neuroscience, Rehabilitation, Ophthalmology, Genetics, Child and Maternal Health (DINOGMI) University of Genoa Genoa Italy; ^8^ Fondazione Policlinico Universitario A. Gemelli IRCCS Rome Italy; ^9^ Università Cattolica del Sacro Cuore Rome Italy; ^10^ Child Neurology and Psychiatric Unit Pediatric Hospital G. Salesi, Azienda Ospedaliero‐Universitaria Delle Marche Ancona Italy; ^11^ IRCCS Istituto Delle Scienze Neurologiche di Bologna, Full Member of the European Reference Network EpiCARE Bologna Italy; ^12^ Neurophysiology Unit and Epilepsy Center, Neuroscience Department Azienda Ospedaliero‐Universitaria di Modena Modena Italy; ^13^ Functional Neurosurgery Unit, Department of Neurosurgery Fondazione IRCCS Istituto Neurologico Carlo Besta Milan Italy; ^14^ Section of Anatomy and Histology, Department of Neurosciences, Biomedicine, and Movement Science, School of Medicine University of Verona Verona Italy; ^15^ Division of Clinical Neurophysiology and Epilepsy Center IRCCS Ospedale Policlinico San Martino Genoa Italy

**Keywords:** Broca, Epilepsy surgery, Language area, MRI, Stereo‐EEG, Werniche

## Abstract

Epilepsy surgery in language areas is challenged by the intricacies of presurgical workup and surgical planning. In recent decades, the view of language‐related circuitry has shifted from being localized in a few cortical centers to a distributed, dynamically interconnected system, increasing complexity. In this framework, neuropsychology, functional neuroimaging and neurophysiological assessments play an essential role in minimizing the risk of language deficits. A comprehensive preoperative neuropsychological assessment is essential for providing a baseline against which to compare postoperative performance, as well as for helping to define functional deficit and epileptogenic zones. Functional magnetic resonance imaging (fMRI) represents a fundamental noninvasive alternative to the traditional Wada test for determining hemispheric language lateralization and localization. Resting state fMRI (rs‐fMRI) may offer a compelling alternative, particularly in populations where task performance is difficult or unreliable. The role of Stereo‐electroencephalography (Stereo‐EEG) and the intracerebral electrical stimulations (ES) is pivotal for both the identification of the epileptogenic zone and the mapping of eloquent cortices. The use of all available cortical stimulation methods, combined with test batteries targeting different language subdomains and designed to avoid the induction of post‐discharges, appears to be the optimal approach for maximizing patient safety. The preservation of language function also relies on highly specific intraoperative mapping techniques, primarily employed in cooperative patients during awake craniotomy. Likewise, cortico‐cortical evoked potentials could represent a valuable “task‐free” neurophysiological alternative. Surgical strategies involving language areas vary from focal resections to larger lobar, multilobar or hemispheric procedures. Minimally invasive options such as Stereo‐EEG‐guided radiofrequency thermocoagulation (RF‐TC) and laser interstitial thermal therapy (LITT) have broadened therapeutic possibilities. Selection depends on EZ accessibility and extent. Modern epilepsy surgery within language‐eloquent areas increasingly relies on integrated multimodal approaches. Future directions include the use of machine learning to analyze large postoperative datasets and predict long‐term functional and seizure outcomes.

**Key points**

1. Epilepsy surgery in language areas is challenged by complex presurgical and surgical workup and risks of complications
2. Recently, the view of language has shifted from being localized in a few cortical centers to a distributed, dynamically interconnected system
3. A comprehensive preoperative neuropsychological assessment is crucial to localize functional deficit zone and epileptogenic zone.
4. BOLD‐fMRI and resting‐state‐fMRI are complementary, indispensable tools for presurgical language evaluation in pediatric epilepsy.
5. Language functions could be studied by analyzing both the Stereo‐EEG signal and the electrical stimulations results at different frequencies.
6. Intermediate‐Frequency Stimulations are comparable as efficacy to high‐frequency stimulation but are less associated with the occurrence of post‐discharges.
7. The preservation of language function relies also on highly specific intraoperative mapping techniques, tailored to age and level of cooperation.
8. Surgical strategies involving language areas vary from focal resections to larger lobar or hemispheric procedures
9. Integration of multimodal approaches is essential to minimize the risks of postoperative deficits while maximizing the chances of seizure freedom.

**ILAE competencies and learning objectives**

**1.5 Accurately order and interpret neuroimaging as it pertains to epilepsy**

***Learning objectives***

1.5.1 Recognize the spectrum of MRI sequences optimized for epilepsy (L2)
1.5.4 Decide when to conduct specialized neuroimaging and which type (e.g., functional, metabolic, post‐processing, etc.) (L2)
1.5.5 Interpret and apply the results of specialized neuroimaging accurately in the clinical context (L3)

**4.3 Demonstrate working knowledge of advanced techniques for presurgical evaluation**

***Learning objectives***

4.3.2 Recommend functional neuroimaging as appropriate (L3)
4.3.3 Recommend and interpret results of neuropsychological testing (L3)
4.3.4 Demonstrate working knowledge on planning implantation of intracerebral electrodes (L3)
4.5 Demonstrate ability to integrate information from multimodal work‐up and estimate risks and benefits of surgical therapy

**4.6 Demonstrate working knowledge of postsurgical follow‐up**

***Learning objectives***

4.6.2 Demonstrate working knowledge of postsurgical neurological, neuropsychological and psychiatric complications (L3)
4.6.4 Identify situations which require rehabilitation following surgery (L3)
4.7 Demonstrate the value and need for multidisciplinary teamwork

## 1 INTRODUCTION

Epilepsy surgery represents the best treatment option for carefully selected patients^1^ with drug‐resistant epilepsy (DRE),^2^, ^3^ yielding satisfactory seizure outcome in most.^1^, ^4^

However, this is one of the most underutilized treatment options for epilepsy, due to several factors, including the fear of cognitive and functional deterioration, especially when surgery involves eloquent brain areas.^5^, ^6^ Surgical treatment in language areas is associated with significant uncertainties regarding optimal presurgical evaluation protocols and possible complications.^7^

In 1959, Wilder Penfield and Lamar Roberts published *Speech and Brain Mechanisms*, a landmark work that included detailed observations from surgical patients. They reported on some of the earliest applications of intraoperative direct brain electrostimulation for language mapping performed in awake conditions. Although preliminary, these findings underscored the pivotal contribution of epilepsy surgery to our understanding of the anatomical and functional organization of language.^8^ Subsequently, this knowledge opened the door to the possibility of operating within highly eloquent cortical regions, by achieving a more favorable balance between postoperative outcomes and postsurgical morbidity. Progressive refinements in epilepsy surgery, including improved preoperative anatomo‐electroclinical correlation, advanced neuroimaging techniques, optimized presurgical planning, and intraoperative neurophysiological monitoring, have significantly enhanced seizure control while reducing the risk of permanent long‐term language deficits.^9^

Key factors influencing surgical strategies include the underlying pathology, the location and extent of the epileptogenic zone (EZ), surgical techniques, intraoperative mapping, and monitoring efficiency.^10^ Common etiologies encompass low‐grade epilepsy‐associated tumors, focal cortical dysplasia, vascular malformations, and post‐ischemic or traumatic lesions.^10^ These are often surgically approachable when distinct from eloquent regions.^11^ In case of insufficient information derived from noninvasive investigations, especially if MRI is unrevealing, Stereo‐electroencephalography (Stereo‐EEG) helps identifying EZ and its relationship with surrounding eloquent cortex and subcortical tracts.^12^

Based on these premises, the Commission of Epilepsy Surgery of the Italian League against Epilepsy (LICE) held a workshop in the context of the 2025 LICE Annual meeting to discuss current knowledge on epilepsy surgery in language areas.

Here we present a summary of that workshop.

## 2 NEUROANATOMY OF LANGUAGE SYSTEMS, BEYOND BROCA AND WERNICKE (FIGURE 1)

**FIGURE 1:**
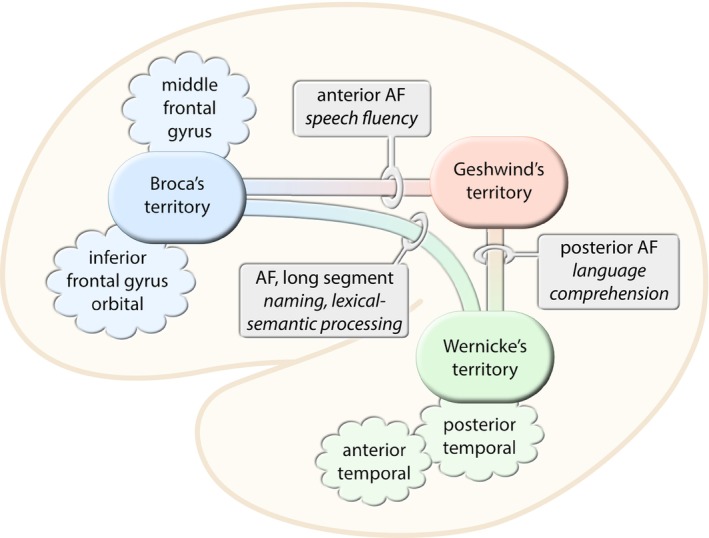
Schematic representation of language areas.

Identifying the neural bases of the language system is foundational not only for understanding one of the most complex cognitive functions in the human brain, but also for developing broader, network‐based models of brain organization. In recent decades, a paradigm shift has occurred, veering from a view of language as a function localized to a few cortical centers toward one based on a distributed, dynamically interconnected system.

The quest to define the neuroanatomical structures responsible for language processing began with the identification of Broca's area in the inferior frontal gyrus and Wernicke's area in the posterior superior temporal gyrus in the mid‐to‐late 19th century.^13^ These discoveries, rooted in postmortem analysis of patients with distinct aphasias, helped establish the concept of functional specialization, associating the former with language production and the latter with comprehension. The classical model relied on the presence of the arcuate fasciculus (AF), a thick white‐matter bundle connecting the two areas, to provide a seemingly simple anatomical circuit for linguistic processing.

However, clinical and imaging studies have long pointed to the limitations of a language theory based solely on highly localized functions. Purely focal lesions rarely produced the textbook aphasia syndromes, and many linguistic functions were shown to engage territories far outside the classic perisylvian boundaries. Multiple sources of uncertainty have been highlighted. First, significant inter‐individual variability in the topography of cortical areas makes it arduous to generalize the assignment of functions to specific cortical territories. Additionally, the brain's inherent ability to re‐route processing following cortical damage is well documented. Indeed, functional properties may, to a certain extent, be transferred to or maintained by other cortical areas, provided that proper long‐range connectivity is available. Language function, therefore, is not an intrinsic property of a cortical patch but an emergent property of the circuit in which that area participates.^14^

In this context, a detailed reconstruction of white‐matter pathways in the perisylvian region is key. Recent advances in noninvasive neuroimaging, particularly diffusion tensor tractography, together with a revival of classical anatomical dissections,^15^ have revealed far more intricate connectivity patterns than previously assumed. For instance, the AF is no longer viewed as a single, homogeneous white‐matter bundle, but rather a composite system with multiple components.^16^ At least three distinct segments provide reciprocal connections to three key perisylvian territories. A long segment forms the direct connection between the posterior temporal cortex (Wernicke's area) and the inferior frontal gyrus (Broca's area). However, the connection is also indirect, involving an intermediary hub in the inferior parietal lobule (often referred to as Geschwind's territory). Specifically, an anterior segment links Broca's area with the inferior parietal lobule, while a posterior segment connects the parietal hub to Wernicke's area. This tripartite organization implies that language relies on both direct and indirect frontotemporal communication, fundamentally redefining the anatomical substrate of language circuitry.

In addition, MRI tractography shows that fiber distribution at each of the target territories is significantly wider than previously thought, supporting a broader and more distributed organization of language networks compared to the classical view.^16^

A substantial contribution to the ongoing revision of the cerebral language system comes from functional imaging studies. Among several lines of investigation, an especially suggestive hypothesis is the dual‐stream model, conceptually analogous to the dorsal (“where”) and ventral (“what”) dichotomy long recognized in the visual system. This framework proposes two partially segregated yet interacting pathways linking temporal and frontal areas.^17^, ^18^, ^19^ The dorsal stream, built on the arcuate and superior longitudinal fasciculi, supports sensorimotor integration, critical for mapping sounds to articulatory representations (i.e., speech production and repetition). Regions belonging to the dorsal stream include the posterior superior temporal gyrus, planum temporale, supramarginal gyrus at the parietal–temporal junction, and the posterior inferior frontal gyrus (Broca's area). The ventral stream, coursing through the extreme capsule fiber system, which includes the inferior fronto‐occipital fasciculus, the uncinate fasciculus, and the inferior longitudinal fasciculus, subserves sound‐to‐meaning mapping and comprehension. The middle and inferior temporal gyri, the anterior temporal lobe, and the ventrolateral prefrontal cortex (including the anterior portions of the inferior frontal gyrus, such as pars triangularis and orbitalis) are considered part of the ventral stream.^17^, ^18^

The dual‐stream model formalized the recognition of language as the result of a distributed network that extends well beyond Broca's and Wernicke's areas, encompassing subcortical structures and even the cerebellum.^20^ High resolution functional imaging studies have since defined a “core language network” as a functionally cohesive, language‐selective system that is distinct from both domain‐general cognition and sensorimotor systems, highlighting the unity of the language function as a whole.^21^

In the context of epilepsy surgery, these modern insights transform the planning of resections near eloquent cortex.^22^ Traditional intraoperative mapping, focused only on identifying cortical loci where stimulation causes speech arrest, is insufficient to delineate the extended perisylvian network. Patient‐specific mapping using functional MRI and diffusion weighted imaging is necessary to reveal the individual organization of both the dorsal (phonological) and ventral (semantic) streams. Crucially, the recognition of network plasticity and interhemispheric reorganization, common in early‐onset epilepsy, underscores that language preservation requires a network‐based approach, rather than a purely focal strategy.^23^

In this framework, neuropsychology, neuroimaging, and both invasive and noninvasive neurophysiological assessments play an essential role in optimizing surgical planning and minimizing the risk of permanent language deficits.

## 3 THE ROLE OF NEUROPSYCHOLOGY

A comprehensive preoperative neuropsychological assessment is crucial for providing a baseline against which to compare postoperative performance, helping to localize and lateralize functional deficit zone and EZ.^24^ In this context, the assessment of language functions is associated with a high degree of complexity and should consider age, cognitive level, and behavioral issues. The core functions to be assessed include language comprehension, verbal expression, naming, repetition, and speech production, corroborated by the evaluation of memory and executive functions.^24^ Ideally, the same assessment protocol should be repeated after surgery to allow for meaningful comparisons. However, this becomes more challenging in children, as developmental milestones achieved between the pre‐ and postoperative assessments must be considered.

In the event of surgical complications, a comprehensive postoperative assessment is crucial for detecting possible language deficits and monitoring function recovery over time. A recent systematic review^25^ demonstrated that most studies reported only short‐term outcomes and less than 50% estimated the reliable change index (RCI) to measure postoperative changes. Confrontation naming was the most frequently tested function (96%), followed by verbal fluency (47%) and language comprehension (13%). It has also been suggested that current approaches to language assessment may underestimate the prevalence of language impairment in epilepsy patients and should include, for example, verb naming and picture description subtests.^26^

Ultimately, to integrate neuropsychological assessment with the results of functional neuroimaging and neurophysiological studies, the involvement of a neuropsychologist is mandatory in developing individualized stimulation paradigms.

## 4 THE ROLE OF NEUROIMAGING

Functional Magnetic Resonance Imaging (fMRI) has emerged as a relevant noninvasive alternative to the traditional Wada test, for determining hemispheric language lateralization and localization of language areas.^27^, ^28^ However, the Wada test may still be considered in selected clinical situations. These include cases with inconclusive or technically limited fMRI results (for example poor task performance or excessive motion), discordant findings between fMRI, neuropsychological assessment, and electroclinical data, or suspected atypical or bilateral language dominance when the results may influence surgical planning.^29^

Task‐based fMRI (tfMRI) fundamentally relies on the blood oxygenation level Dependent (BOLD) signal, which indirectly measures neuronal activity through neurovascular coupling.^30^ This mechanism involves a transient increase in local microvascular blood flow that exceeds oxygen consumption in active brain regions, resulting in an excess of oxygenated hemoglobin and a reduction in deoxygenated hemoglobin, which is detected as a BOLD signal change.^30^ In the context of epilepsy, tfMRI is widely utilized to map eloquent brain regions, especially those involved in language and motor functions. However, this technique presents unique challenges in pediatric patients due to the relatively high prevalence of atypical language representation^7^ (ranging from 20% to 50% in epilepsy patients compared to 2.5% in healthy controls), frequently co‐occurring cognitive impairment or behavioral disorders, and the high variability of paradigms used across centers.^28^

Language task design is pivotal, yet clinical practice across Europe lacks standardization.^27^, ^31^ Common paradigms include phonemic verbal fluency and auditory comprehension to identify inferior frontal and superior temporal language territories, respectively.^27^ Other effective tasks include word generation (phonemic decision, semantic verbal fluency, verb generation), which robustly activates frontal gyri, and semantic decision paradigms (sentence completion, verb‐to‐noun generation), which can activate both inferior frontal and superior temporal gyri.^27^, ^28^ In children, adaptive tasks and passive listening (e.g., passive story listening) are particularly valuable, especially under age 4 or with cognitive impairment, when active tasks are unreliable. Many advanced neuroimaging centers train patients before scanning and adapt tasks to cognitive status, yet dedicated paradigms for cognitively impaired patients remain uncommon.^27^

The interpretation of BOLD‐fMRI language maps requires careful consideration due to their high sensitivity but potentially low specificity, which can lead to redundant activations.^28^

Additionally, BOLD activations identify regions involved in language processing, but these do not necessarily correspond to cortical sites that are essential for long‐term language function. Some activated regions may represent supportive or redundant components of the language network. Postoperative language disturbances may therefore be transient and reflect network reorganization rather than permanent loss of function.

The laterality index, which quantifies the relative activation difference between the left and right hemispheres, is a common metric, but thresholds and signal variability can influence its calculation.^28^ Atypical language lateralization is more common in epilepsy patients, particularly those with earlier seizure onset or specific lesion types.^28^ The American Society of Functional Neuroradiology recommends using at least two paradigms for presurgical language lateralization to capture the complexity of language function and account for inter and intrahemispheric reorganization in people with epilepsy.^27^ Clinical interpretation aims to prevent postoperative language impairment while ensuring effective seizure control, recognizing the risks of both false positive and false negative fMRI activations. Qualitative visual inspection is the primary method for interpreting lateralization, with a minority also using quantitative analysis.^27^ The predictive value of tfMRI for postsurgical cognitive outcomes in left temporal lobe epilepsy is modest, influenced by factors such as sex, education level, age of seizure onset, and disease duration.^32^ The clinical impact of preoperative language fMRI may vary according to the clinical scenario. In patients with lesions close to language areas who are already candidates for awake craniotomy or invasive functional mapping, language fMRI may not significantly alter surgical strategy, as direct cortical stimulation remains the reference standard for defining indispensable cortex. In these cases, fMRI may still offer valuable preliminary insights into language lateralization and can help guide electrode implantation strategies as well as intraoperative mapping.

Language fMRI may play a more relevant role in patients in whom invasive mapping is not feasible, in those with suspected atypical language representation (e.g., early‐onset epilepsy), or when neuropsychological, clinical and electroclinical findings are discordant. In typical right‐handed patients with left temporal lobe epilepsy undergoing anterior temporal lobectomy, language fMRI findings often confirm expected left‐hemispheric dominance and may not substantially alter the surgical approach. However, when interpreted alongside neuropsychological assessment and structural imaging, they can contribute to estimating the risk of postoperative language decline within a multimodal presurgical evaluation.

Resting‐state fMRI (rs‐fMRI) offers a compelling alternative, particularly in populations where task performance is difficult or unreliable, such as young children or those with significant cognitive deficits.^32^ Instead of active tasks, rs‐fMRI measures spontaneous, low‐frequency BOLD signal fluctuations (0.01–0.10 Hz) that occur while a subject is at rest, which reflect intrinsic functional connectivity (FC) and the organization of brain networks.^33^, ^34^ This method, less demanding for patients, does not require explicit task compliance, and the BOLD signal fidelity remains high even under anesthesia, a common practice in pediatric imaging.^34^

Resting‐state fMRI analyses commonly use independent component analysis (ICA) to delineate large‐scale cortical networks, including language, visual, somatomotor, and default mode networks.^33^, ^34^ In epilepsy, Resting state networks (RSNs) are altered with reduced functional connectivity within and between networks, implicating epileptic activity in baseline network reorganization.^33^ Focal cortical dysplasia–related epilepsy is often associated with global disruption of cortical functional networks, and FCD pathological subtypes can shape these patterns.^33^ rs‐fMRI in pediatric epilepsy surgery has the potential to predict postsurgical seizure and cognitive outcomes.^35^ Greater similarity of resting‐state FC to healthy controls predicts better language outcomes after surgery.^33^ “More normal” connectivity within resting‐state language networks is associated with better overall language outcomes, whereas stronger connectivity on the epileptogenic side has been linked to greater postsurgical memory decline.^33^

Nonetheless, patient motion and the lack of pediatric standardized functional network maps remain major pitfalls that may complicate the interpretation of results.^33^, ^34^

In conclusion, BOLD‐fMRI and rs‐fMRI represent useful and complementary tools for presurgical language evaluation in pediatric epilepsy (Figure 1). Task‐based fMRI provides task‐specific localization and lateralization, while rs‐fMRI offers a noninvasive, task‐independent assessment of functional network organization, which may be particularly useful in non‐compliant or cognitively impaired children.^33^, ^34^ When integrated with structural imaging, neuropsychological assessment, and electrophysiological data, these approaches may contribute to a more comprehensive presurgical evaluation in patients with focal epilepsies. Further standardization of acquisition protocols, analysis methods, and reporting is needed to improve clinical applicability and inter‐center comparability in the developing brain.^28^, ^33^

Diffusion tensor imaging (DTI) and tractography represent critical MR tools capable of integrating fMRI results by providing the essential structural substrate for functional activation.^36^ While fMRI maps dispersed gray matter regions involved in language processing, DTI reveals the underlying white‐matter connectivity required for the synchronized parallel processing of this network.^36^ This noninvasive technique works by estimating the anisotropic movement of water molecules along parallel axons to reconstruct the brain's structural connectome.^37^ Quantitative metrics derived from DTI, such as fractional anisotropy (FA) and mean diffusivity (MD), allow clinicians to characterize microstructural integrity, where a decrease in FA often indicates damage.^36^, ^37^

The clinical utility of this tool hinges on a structural resolution limit, where the voxel size must be sufficiently small to resolve the anatomical geometry of interest.^38^ Standard DTI often faces challenges in “bottleneck” regions due to crossing fibers, which occur in 70%–90% of voxels and can cause erroneous diffusion metrics through partial volume effects.^37^, ^38^ Furthermore, substantial variability exists across centers in acquisition protocols, reconstruction algorithms, and post‐processing pipelines, which may limit direct comparability of tractography results. Consequently, tractography is generally interpreted as a probabilistic anatomical visualization tool and is typically integrated with structural and functional imaging, as well as intraoperative mapping, as part of a multimodal presurgical assessment.

To overcome technical limitations, advanced methods like Fiber Orientation Distribution (FOD) or constrained spherical deconvolution (CSD) are utilized to resolve multiple fiber directions within a single voxel.^37^, ^39^, ^40^ While lower spatial resolutions can improve the signal‐to‐noise ratio and reduce path length dependency, higher resolutions (i.e., 1.5 mm isotropic) now allow for the precise examination of smaller structures like short association fibers.^37^

Such advancements are essential for visualizing the arcuate fasciculus, which connects perisylvian regions involved in syntax and word learning, as well as the uncinate fasciculus and inferior longitudinal fasciculus, which is essential for lexical retrieval.^37^ Preoperative tractography may support visualization of patient‐specific white‐matter anatomy and assist surgical planning, particularly in dominant temporal lobe procedures. In clinical practice, its main contribution consists in delineating the relationship between the epileptogenic zone (EZ) and major language‐related white‐matter tracts, thereby helping to estimate potential surgical risks. However, its predictive value for individual postoperative language outcome remains limited,^36^ and it should be regarded as a complementary planning tool rather than a substitute for intraoperative white‐matter stimulation.^37^

## 5 THE ROLE OF STEREO‐ELECTROENCEPHALOGRAPHY (STEREO‐EEG)

Both Stereo‐EEG and subdural electrodes (SDE) are well‐established invasive techniques for the presurgical evaluation of patients with focal drug‐resistant epilepsy, enabling the identification of the EZ and the functional mapping of eloquent cortex, including language areas.

Subdural grids and strips provide two‐dimensional, high‐density coverage of the cortical surface. This configuration enables extensive sampling of the gyral cortex and systematic functional mapping via electrical stimulation of adjacent leads. However, SDE implantation requires a craniotomy, making the procedure more invasive and potentially associated with higher morbidity. Additionally, SDE primarily samples the cortical surface, providing limited access to sulcal regions, deep cortical structures, and subcortical pathways, which are increasingly recognized as essential components of the language network.^41^, ^42^

In contrast, Stereo‐EEG employs stereotactically implanted depth electrodes, enabling three‐dimensional exploration of both cortical and subcortical structures (e.g., deep opercular regions, cingulum, insula, and white‐matter pathways). This approach facilitates investigation of distributed language networks and is particularly advantageous in cases involving deep EZ or complex anatomo‐functional reorganization. Stereo‐EEG implantation is less invasive, as it avoids craniotomies, and is associated with improved patient tolerability and shorter recovery times.^41^, ^42^

Although Stereo‐EEG provides a more limited sampling of the cortical surface—since electrode placement is hypothesis‐driven and restricted to predefined trajectories—it has become the preferred invasive technique in many comprehensive epilepsy centers. Its superior ability to explore cortical regions both at the surface and within deeper structures, combined with a more favorable safety profile compared with SDE, makes it ideal for epileptological study and functional mapping.^41^, ^43^

For these reasons, we focus on the use of Stereo‐EEG, particularly through intracerebral electrical stimulation (ES), for the investigation and mapping of language areas.^41^, ^44^

During the presurgical workup in focal, drug‐resistant epilepsies, Stereo‐EEG and ES are pivotal both for localizing the EZ and for mapping eloquent cortex.^43^, ^45^, ^46^

The strategy of implantation, strictly tailored for each patient on the basis of presurgical workup,^43^, ^45^ provides access to all the cortical areas, allowing a three‐dimensional definition of the spatial and temporal organization of the EZ.^45^ The electrodes are positioned based on assumptions regarding the location of the EZ, and similarly, the decision as to which language areas need to be explored is guided by the preliminary and previous epileptological study.^45^

Primary functional areas such as sensorimotor, auditory, or visual cortex can be reliably identified with ES, given their relatively consistent anatomical localization and ease of testing.^47^

The mapping of language areas (LAs) might be more challenging, as the precise localization and extent of LAs depend on individual variability, particularly in patients with epilepsy, with a substantial variability across individuals. ^47^, ^48^, ^49^Right hemispheric dominance, bilateral patterns or unusual left localization, are reported in up to one third of patients with focal epilepsyi.^50^, ^51^

Stereo‐EEG planning aims to acquire the most comprehensive dataset possible. Therefore, a comprehensive presurgical workup is essential to ensure adequate exploration of the different cortical areas, given the intrinsic limitations of the Stereo‐EEG technique, as electrode placement determines the information that can be obtained and restricts it to the cortical regions sampled.^46^, ^52^

Thus, the implantation strategy should carefully consider the possible modifications of the language network in cortical malformations or other structural abnormalities, the activated areas during language functional MRI, and any clinical data potentially helpful as handedness, any language disorders or delays, ictal and postictal language deficit. These data might be very helpful to plan the Stereo‐EEG and verify the localization and lateralization of language functions, considering that in some cases a bilateral Stereo‐EEG and alternative trajectories for covering the hypothetical functional areas are highly recommended (see Clinical case 1).

Language functions can be studied through both passive analysis of Stereo‐EEG signals and ES results, integrated with fMRI and clinical data.

Intracerebral EEG signal analysis consists of passive activation, allowing for the site‐specific identification of the cortical sites involved in language processing. Signals are acquired for analysis during repetitive trials in specific tasks (naming, reading, comprehension), segmented into epochs aligned with administered trials, and finally analyzed, assessing possible statistical significance. ^53^Results are highly dependent on the patient's cooperation in the individual tests.

On the contrary, ES likely act by temporarily interfering with linguistic functions, causing a more or less evident dysfunction. An appropriate testing environment for the assessment of various language functions is essential and should be tailored to the patient's age and cognitive level.

The effectiveness of low‐frequency (1 Hz, LFS) and high‐frequency stimulations (50 Hz, HFS) in assessing eloquent areas is well established.^54^, ^55^ As reported by Trébuchon,^54^ HFS (phase duration 1 msec, current intensities 0.2–2.5 mA, duration 3–5 s) are effective in determining the site and extent of LAs. Increasing the current intensity of electrical stimulations can amplify the sensitivity of the method, but also the post‐discharges (PD), which instead reduce the possibility of LAs localization. HFSs employ a duration of few seconds (3–7 s) and a variable current intensity, ranging from 0.2 to 5 mA.^56^ This protocol may limit the possibility of testing patients and of identifying any deficits in the various areas of linguistic function.

**FIGURE 2:**
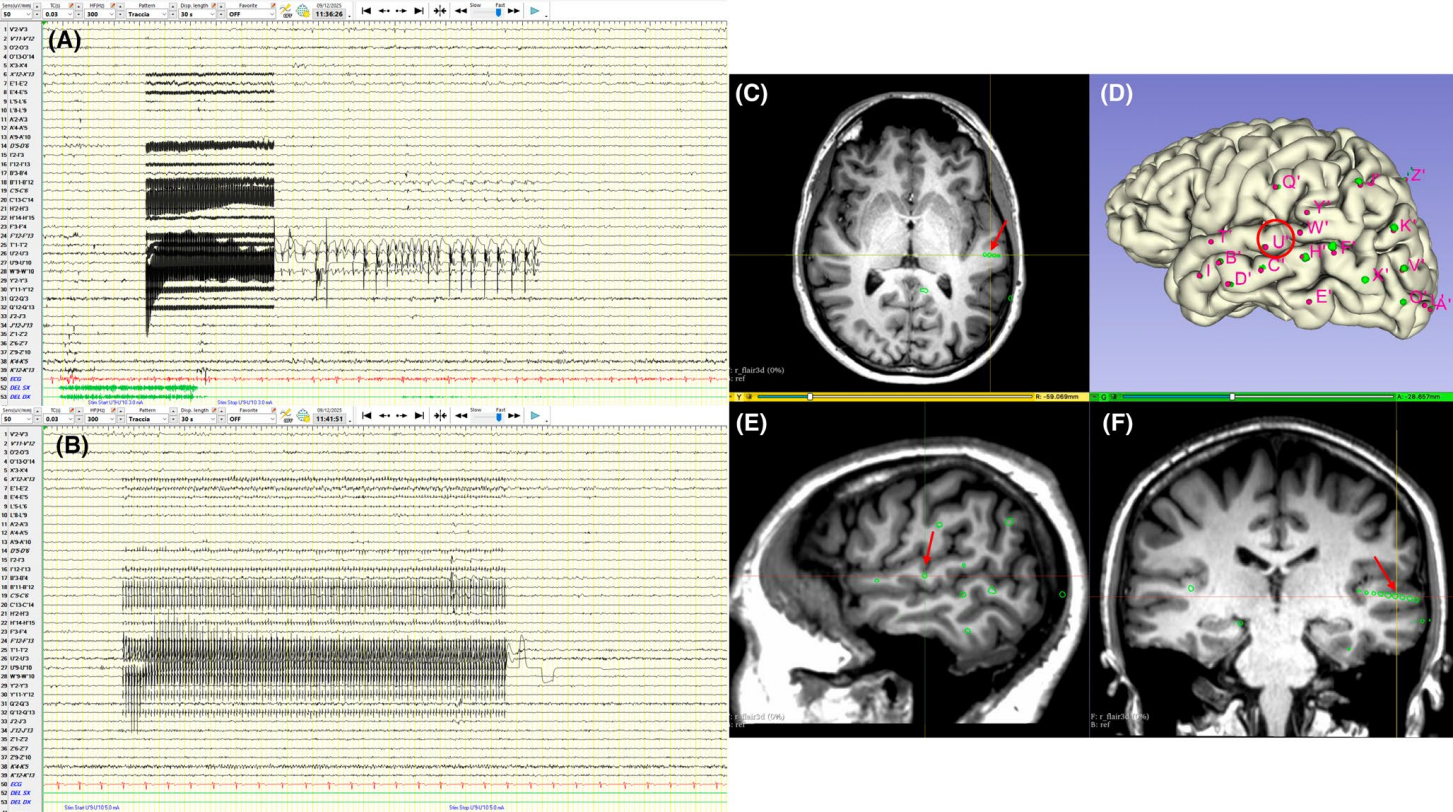
Comparison between intracerebral electrical stimulations at 50 Hz and 9 Hz in language areas. (A) Intracerebral electrical stimulation at 50 Hz (frequency), 3 mA (current intensity), 0.5 ms (pulse width), and 5 s (duration), delivered through contacts U'9–U'10 (mid‐posterior superior temporal gyrus). A post‐discharge lasting approximately 10 s is observed on contacts exploring the superior temporal gyrus (T', W', U'), with less pronounced activity on contacts exploring the middle temporal gyrus (B' and C'). In both stimulation we obtained a positive clinical response: During 50 Hz stimulation, the patient was reading, then stopped, and subsequently was unable to follow simple commands. (B) Intracerebral electrical stimulation at 9 Hz (frequency), 5 mA (current intensity), 0.5 ms (pulse width), and 15 s (duration), delivered through contacts U'9–U'10 (mid‐posterior superior temporal gyrus). No post‐discharge is observed. During 9 Hz stimulation, a specific comprehension protocol was administered, involving the reading of predefined sentences and asking the patient to indicate whether they were true or false. The patient was unable to understand the meaning of any of the sentences. (C, E, F) Multiplanar representation of a T1‐weighted 3D sequence in the axial (C), sagittal (E), and coronal (F) planes. The exact position of contacts U'9–U'10 is indicated by arrows. (D) Three‐dimensional pial surface. The position of electrode U' is indicated by a circle.

To overcome these limitations, intermediate‐frequency stimulation (IFS; 6–15 Hz) has been proposed.^57^ IFS uses a variable stimulation frequency based on clinical response while keeping current intensity (5 mA), duration (15 s), and pulse width (0.5 msec) constant. IFS has efficacy comparable to HFS but induces fewer PDs, allowing longer stimulation times and more extensive language testing^52^, ^53^ (Figure 2).

While patient management during Stereo‐EEG may differ across centers and clinicians, this flow chart provides a practical framework for IFS in language mapping:

1. Selection of contacts: Electrode sites within or adjacent to hypothesized language networks are chosen based on clinical, anatomo‐electroclinical, and fMRI data.
2. Anti‐seizure medications must be stable and, if previously reduced, must be restored to baseline levels.
3. Time efficiency: HFS are performed initially; if they elicit a clinical effect attributable to a language disturbance, IFS are subsequently administered.
4. Stimulation initiation: Start at 6 Hz, 5 mA, 0.5 msec pulse width, 15 s duration. Continuous Stereo‐EEG monitoring is mandatory to detect post‐discharges (PD).
5. Task performance: During stimulation, the patient performs a standardized language task targeting the relevant domain (e.g., naming, reading, comprehension). The task consists of a variable number of trials, depending on the specific paradigm, but with the aim to ensure an adequate number of trials administered during stimulation. Multiple blocks of the same task may be administered.
6. Frequency: If no deficit or PD occur, frequency is progressively increased (e.g., 9, 12, up to 15 Hz) while maintaining intensity and duration, aiming to elicit reproducible clinical effects. When a clinically significant response is obtained (e.g., speech arrest, anomia, phonemic or semantic paraphasias, comprehension errors), it is no longer necessary to increase the stimulation frequency.
7. PD management: If PD occur, the frequency may be reduced, or the contact excluded from further testing.

For this type of stimulations to be effective and reliable, the patient's full cooperation is required.

Table 1 provides a concise illustrative guide for centers wishing to expand their use of intracerebral electrical stimulation during Stereo‐EEG. However, the implementation of stimulation protocol depends on several factors, including the center's experience, the type of patients treated (e.g., adults vs. pediatric patients), and the patient's epilepsy profile (e.g., epileptogenic zone involvement of language areas, unrevealing brain MRI findings, or imaging suggestive of cortical malformations).

**TABLE 1:** Proposed protocol for LAs stimulation during Stereo‐EEG.^46^, ^47^, ^57^

	Object	Data/tools	Task	Response
Step 1	Selection of contacts to be stimulated	On the basis of clinical, anatomical, fMRI data		
Step 2	Verification of the presence of the function	High‐frequency Stimulations (at least 2 or 3 mA)	Reading a list of words with arms elevated	Assessment of a clinically relevant response, such as errors of varying severity in reading
Step 3 (if results clinically significant in step 2)	Language study	Intermediate‐Frequency Stimulations (6‐15 Hz, 5 mA, 15 s)	Language task targeting the relevant domain (Naming, Reading, Comprehension)	Assessment of a clinically relevant responses, such as errors of varying severity in reading, comprehension, or naming
Step 4	Language study	Modulate the frequency (increasing or reducing) based on clinical response and/or presence of PD	Language task targeting the relevant domain (Naming, Reading, Comprehension)	Assessment of a clinically relevant response, such as errors of varying severity in reading, comprehension, or naming

Abbreviation: LAs, language areas.

In conclusion, in the management of Stereo‐EEG, an in‐depth assessment of language functions using all available stimulation techniques, combined with task batteries targeting multiple language subdomains and designed to minimize PD, provides the information necessary for safe and effective surgical planning in or near language areas with positive implications for the patient's postsurgical outcome.

### 5.1 The role of intraoperative neurophysiological monitoring (IOM) and TMS

The primary objective of Intraoperative Neurophysiological Monitoring (IOM) is to optimize the extent of resection/disconnection of the Epileptogenic Zone, while rigorously protecting against permanent neurological deficits. IOM methodologies are broadly categorized into Testing/Mapping, which aims to functionally identify eloquent nervous structures—such as motor and language areas or deep pyramidal tracts—and monitoring, which provides continuous assessment of the functional integrity of various neural systems, including motor and sensory pathways. The preservation of language function relies on highly specific intraoperative mapping techniques, primarily employed during awake craniotomy, the gold standard in adult and cooperative patients.^58^ The foundational technique for intraoperative cortical mapping of language areas is represented by the Direct Electric Stimulation (DES^58^) and, specifically, the low‐frequency bipolar stimulation (50 Hz in Europe, 60 Hz in North America). This method creates a focused bipolar electric field and a homogeneous current density, where the anodic current can enhance stimulation effects at lower intensities^59^, ^60^ and is still considered the gold standard for example during awake surgery.^61^

However, the broad utilization of IOM faces limitations and challenges, as application in pediatric neurosurgery. The literature is sparse, clear normative data and standardized IOM protocols are lacking, and the spectrum of pathologies differs from that of adults, with a higher frequency of focal cortical dysplasias, including extratemporal locations.^10^

Moreover, concomitant syndromic conditions further complicate IOM interpretation.^62^Regarding anesthetic management, protocols must account for the increased sensitivity of younger children to volatile agents^63^.The use of awake craniotomy is significantly restricted in children under 10^55^years old, due to their limited capacity for sustained collaboration^64^. Furthermore, the functional localization of language areas shows variability with age,^65^ and consequently, there might be greater reliance on extraoperative mapping techniques, such as Stereo‐EEG or subdural grids.^57^, ^66^, ^67^

A valuable “task‐free” neurophysiological alternative is Cortico‐Cortical Evoked Potentials (CCEPs), which offer an assessment of the connectivity between anterior and posterior language areas, specifically tracking the dorsal language pathway via the arcuate fasciculus.^68^, ^69^ The procedure involves low‐frequency bipolar single pulse stimulation (_~_1 Hz, 15–30 mA), generating a characteristic four‐peak waveform (P1, N1, P2, N2). While applicable in anesthetized patients during epilepsy surgery, the success rate under general anesthesia (55.5%–86.2%) remains lower than in the awake setting (92.3%).^70^, ^71^ Challenges include the absence of standardized protocols and signal fluctuations influenced by individual brain anatomy and the depth of anesthesia.^72^Nevertheless, CCEPs may provide prognostic value: the preservation of the CCEP signal is associated with language recovery within 3 months, even if a temporary postoperative deficit occurs.^73^ Specifically, a 50% reduction in N1 amplitude has been proposed as the critical threshold for predicting the preservation of the dorsal language pathway.^74^

Finally, more recently, repetitive transcranial magnetic stimulation (rTMS) has been employed preoperatively to create a temporary “virtual lesion” on the putative language cortex using repetitive 5 Hz pulses.^75^ This method boasts a high negative predictive value (99%–100%) compared to cortical stimulation, making it a strong indicator for diagnostic language mapping.^75^, ^76^ In pediatrics, functional thresholds for TMS are inversely related to age, meaning younger patients often have higher thresholds.^76^ Despite challenges like attention fluctuation, rTMS may represent a feasible technique in children, with a low risk of seizures (0.2%).^77^ However, the use of rTMS to assess language function is limited by its low sensitivity and specificity, poor reliability, and possible stimulation‐associated discomfort, with comparatively little supporting evidence relative to other techniques^78^

## 6 SURGICAL TECHNIQUES AND SEIZURE AND FUNCTIONAL OUTCOME AFTER EPILEPSY SURGERY

Surgical strategies vary from focal resections to larger lobar or hemispheric procedures. Minimally invasive options such as Stereo‐EEG guided radiofrequency thermocoagulation (RF‐TC) and laser interstitial thermal therapy (LITT) have broadened therapeutic possibilities. RF‐TC creates focal thermal lesions through implanted electrodes, whereas LITT delivers MRI‐guided laser ablation with real‐time thermometry, minimizing collateral damage. Selection depends on EZ accessibility and extent.^79^

### 6.1 Temporal lobe surgery

Temporal lobectomy remains the most frequent type of resective surgery for drug‐resistant epilepsy in adolescents and adults. Nonetheless, several studies have reported a non‐negligible incidence of long‐term language impairments following left temporal lobectomy, with rates ranging from 41% to 51% at 6 months and 21%–34% at 1 year—particularly affecting naming, word finding, and verbal fluency. As a result, predictors of postoperative language decline have been extensively studied through a network‐based rather than purely localizationist framework^25^, ^80^, ^81^, ^82^, ^83^

In children, language maturation is often delayed by epilepsy itself. Left‐hemispheric surgery can further slow the progress, particularly in expressive vocabulary, but improvement usually stabilizes within 2 year^84^s

Lesion–symptom mapping identifies the fusiform gyrus and posterior dominant inferior temporal lobe (4–6 cm from the temporal tip) as critical for naming and reading^83^. Nevertheless, simply limiting the posterior resection margin is insufficient to preserve functions, underscoring the need to consider individual variability and dynamic network interactions.^85^, ^86^

Connectivity studies have revealed strong correlations between integrity of white‐matter (WM) pathways and language outcomes. Damage to the arcuate fascicle and the inferior fronto‐occipital fascicle correlates with naming deficits after dominant hemisphere resections, while the middle longitudinal fascicle damage is more relevant in the non‐dominant hemisphere.^37^ Functional MRI analyses further show that postoperative outcomes depend on multiple factors, including surgical side, hemispheric dominance, preoperative network organization, and seizure outcome. Atypical (bilateral or right) dominance often predicts better postoperative fluency in left TLE, whereas typical left dominance favors semantic performance after right TLE. Persistent postoperative seizures are consistently associated with naming deterioration.^87^

Minimally invasive temporal approaches have further improved cognitive preservation. RF‐TCs performed via Stereo‐EEG electrodes show stable seizure control and preserved cognitive function regardless of lesion number.^88^ LITT, while offering lower seizure‐free rates than anterior temporal lobectomy—around 60% after 1 year—produces significantly fewer and milder declines in language, memory and recognition, particularly when the EZ spares lateral and basal temporal regions.^89^, ^90^ Consequently, LITT represents a valuable option in patients prioritizing cognitive preservation, while open resection remains the treatment of choice for extensive or LITT‐refractory epilepsy.^89^

### 6.2 Extratemporal surgery

Language outcomes following extratemporal epilepsy surgery remain less clearly defined, largely due to the lower incidence of such cases and heterogeneity in study design. Frontal, parietal, and insular surgeries show variable effects on language, often influenced by cognitive–executive integration rather than pure linguistic mechanisms.

In frontal lobe epilepsy, research mainly focuses on naming and verbal fluency. Some studies suggest higher baseline language ability predicts greater postoperative decline, although findings remain inconsistent. Deficits often arise from disruption of executive–linguistic networks rather than direct lesion of language areas and may not correspond to classical dominance patterns.^91^, ^92^, ^93^, ^94^

In parietal lobe resections, postoperative language deficits have been reported in fewer than half of the patients, occurring regardless of language dominance, probably reflecting a general baseline limitation in language abilities independent of hemispheric specialization, although the possibility of preoperative cortical reorganization should also be considered, given the chronicity of epilepsy in many surgical candidates.^95^

Resections in the operculo‐insular region are particularly complex due to its extensive connectivity with both frontal and temporal speech systems. Transient dysarthria, naming difficulties, and verbal fluency impairments are common, especially after dominant hemisphere surgery, though long‐term outcomes are usually favorable.^96^

In a heterogeneous pediatric cohort undergoing epilepsy surgery across different etiologies and anatomical sites, long‐term language outcomes did not differ significantly by location or technique. Instead, factors such as baseline cognitive functioning and seizure freedom emerged as stronger predictors of postoperative language trajectories. These findings suggest that, beyond localization, neurodevelopmental plasticity and the functional integrity of language networks play crucial roles in determining outcome.^97^

LITT, guided by diffusion tractography, has also been successfully applied to extratemporal epilepsies near the eloquent cortex. By precisely ablating epileptic tissue while sparing neighboring WM tracts, it offers a promising balance between seizure control and functional preservation.^98^

### 6.3 Disconnections

Disconnection techniques have evolved as an alternative to large resections in cases involving extensive epileptogenic territories by interrupting pathological networks, minimizing tissue removal; they reduce operative time, blood loss, and postoperative complications without compromising seizure control.^99^

For dominant hemisphere anterior disconnections, preserving the pars opercularis and the pars triangularis is critical to maintain language function.^100^, ^101^ Modern multimodal analyses using lesion–symptom mapping and tractography demonstrate that isolating epileptic activity from the broader language network, through callosal or perisylvian disconnection, can facilitate postoperative recovery of language regardless of lateralization.^70^

Posterior quadrant disconnections have achieved excellent seizure outcomes with generally preserved comprehension and expression, sometimes even improving language performance through the reduction of epileptic interference.^102^, ^103^ Rizzi et al. described four surgical variants—full or partial temporo‐parieto‐occipital and temporo‐occipital disconnections—tailored according to the proximity of language‐eloquent posterior cortex.^103^

Hemispherotomy represents the most extensive form of disconnection, reserved for severe unilateral epileptic encephalopathies.^104^, ^105^ Although seizure and complication outcomes are well documented, functional results have been less frequently assessed. A systematic review of language outcomes after hemispherotomy^106^ found impaired language skills in the left surgery group and borderline scores in the right surgery group. Children with cortical dysplasia showed the worst outcomes irrespective of surgery side, while those with left vascular etiology and right‐sided Rasmussen syndrome had the best postoperative performance. A recent large pediatric multicenter study identified earlier epilepsy onset, contralateral hemisphere involvement, polymicrogyria, and persistence on ASM as predictors of worse postoperative developmental outcome after hemispherotomy.^107^ These parameters may guide appropriate patient selection and timing for surgery.

## 7 CONCLUSIONS AND PERSPECTIVES

**FIGURE 3:**
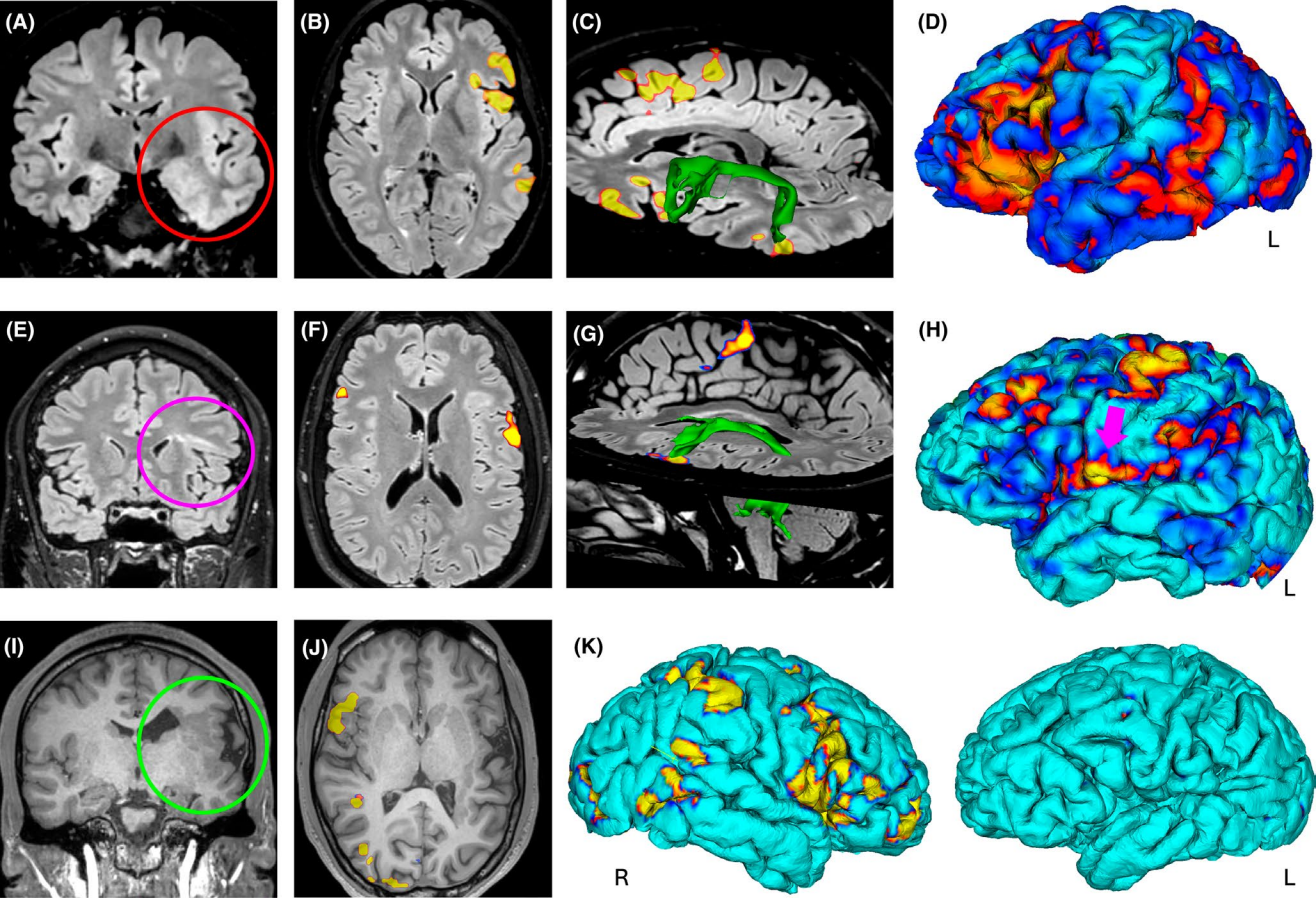
Multimodal Imaging of Language Networks for Epilepsy Surgery Planning (A) 32‐year‐old woman with left temporal lobe epilepsy. Axial FLAIR showing mild hyperintensity of the left anterior temporal pole (red circle). (B) Verbal fluency task‐based fMRI overlaid on FLAIR demonstrates typical left‐hemispheric language lateralization. (C) Tractography‐based 3D reconstruction of the left arcuate fasciculus. (D) 3D pial surface reconstruction with fMRI activations projected onto the cortex, illustrating a typical language network pattern, supporting standard surgical risk assessment. (E) 25‐year‐old man with left frontal focal cortical dysplasia adjacent to Broca's area. FLAIR image showing the dysplastic lesion (violet circle). (F) Phonemic fluency fMRI overlaid on FLAIR reveals local posterior reorganization of the anterior language stream relative to the lesion. (G) 3D tractography of the arcuate fasciculus shows close anatomical proximity to the reorganized activation. (H) 3D pial reconstruction confirms a posterior shift of language activation (violet arrow), directly informing surgical boundaries to minimize language deficits. (I) 9‐year‐old girl with epilepsy due to extensive left‐hemispheric malformation. 3D T1‐weighted MRI demonstrates widespread cortical malformation with heterotopia and polymicrogyria (green circle). (J) Sentence completion fMRI overlaid on 3D T1 shows complete right‐hemispheric reorganization of anterior and posterior language networks. (K) 3D pial surface reconstruction confirms right‐sided language dominance, suggesting a lower expected language risk for left‐hemispheric surgical intervention.

Modern epilepsy surgery within language‐eloquent areas increasingly relies on integrated multimodal approaches (Figure 3). Recent studies integrating structural and functional connectivity with lesion mapping have further refined understanding of language outcomes after surgery, helping delineate at risk regions and compensatory pathways.^108^ Rather than focusing on isolated cortical sites, contemporary models adopt a meta‐network perspective emphasizing the dynamic, distributed, and plastic nature of language organization. This paradigm supports personalized surgical planning, minimizing deficits while maximizing seizure control.

Future directions include the use of artificial intelligence and machine learning to analyze large postoperative datasets and predict long‐term functional and seizure outcomes. Such predictive models may eventually guide individualized risk–benefit assessments and optimize surgical strategies, ultimately prompting early surgery and improving seizure, functional outcomes, and quality of life.

## AUTHOR CONTRIBUTIONS

**Carmen Barba**, **Laura Tassi**: conceptualization and supervision, original paper drafting and review for intellectual content. **Alessandro De Benedictis, Veronica Pelliccia, Domenico Tortora, Federico Melani, Alice Noris, Giuseppe Bertini**: original paper drafting. **Alessandro Consales, Domenica Immacolata Battaglia, Elisabetta Cesaroni, Lorenzo Ferri, Matteo Pugnaghi, Michele Rizzi, Flavio Villani**: review for intellectual content.

## FUNDING INFORMATION

This study has not received any funding.

## CONFLICT OF INTEREST STATEMENT

None of the authors have any conflicts of interest to disclose.

**Test yourself**

1. The most important white‐matter pathways correlated with postoperative poor language outcome are:
  - arcuate fascicle and inferior fronto‐occipital fascicle regardless of hemispheric dominance
  - arcuate fascicle after dominant hemisphere resections and inferior fronto‐occipital fascicle after non‐dominant resections
  - arcuate fascicle and inferior fronto‐occipital fascicle after dominant hemisphere resections
  - middle longitudinal fascicle damage after dominant hemisphere resections
2. Language outcome after hemispherotomy procedure:
  - is positively influenced by long‐lasting seizure history
  - is positively influenced by bilateral preoperative contralateral functional rearrangement
  - does not depend on bilateral hemispheric involvement
  - is negatively influenced by older age at surgery
3. Does stereo‐EEG allow for accurate definition of Language Areas(Las)?
  - No, stereo‐EEG is not useful for mapping language that must be performed in awake surgery.
  - Yes, but only if the electrode implant explores the LAs in both hemispheres.
  - No, it is better to use a mixed implant that includes intracerebral electrodes and grids.
  - Yes, if the implant is guided by anatomical, electroclinical data, cognitive assessment, and fMRI.
4. Which kind of Intracerebral Electrical Stimulation frequencies appear to be optimal for studying linguistic functions?
  - Intermediate frequencies (3–15 Hz) appear to be as effective as High Frequencies but allow for longer stimulation times and fewer percentage of post‐discharges.
  - No type of Intracerebral Electrical Stimulation can perform accurate mapping of LAs.
  - C: Only ES conducted with HF stimulations can help define the location and extent of LAs.
  - D: Low‐frequency stimulations are ideal for studying LAs.
5. What are the most frequently explored language functions across surgical series?
  - Reading abilities and language comprehension
  - Confrontation naming, verbal fluency and language comprehension
  - Verbal fluency and reading abilities
  - Phonemic and Semantic Verbal Fluency tests
6. How are IOM methodologies broadly categorized?
  - Testing and Mapping eloquent areas
  - Motor and sensory pathways monitoring
  - a and b
  - none of the answers is correct
7. Which statement best explains the physiological basis of the BOLD signal used in task‐based fMRI (tfMRI)?
  - It directly measures neuronal firing rates through changes in glucose metabolism.
  - It reflects increased oxygen consumption that exceeds local blood flow in active regions.
  - It results from a mismatch where increased cerebral blood flow exceeds oxygen consumption, leading to reduced deoxygenated hemoglobin.
  - It measures spontaneous low‐frequency signal fluctuations during rest.
8. What is a key advantage of resting‐state fMRI (rs‐fMRI) over task‐based fMRI in epilepsy patients?
  - It provides higher spatial specificity for language localization than tfMRI.
  - It allows functional network assessment without requiring active task performance, even under anesthesia.
  - It eliminates the influence of neurovascular coupling on signal interpretation.
  - It is unaffected by patient motion and does not require post‐processing methods such as ICA.
9. What does the dual‐stream model represent?
  - It is completely different from the dorsal (“where”) and ventral (“what”) dichotomy in the visual system.
  - This framework proposes two interacting pathways linking temporal and occipital areas
  - This framework proposes two interacting pathways linking temporal and occipital areas
  - This framework proposes two interacting pathways linking temporal and frontal areas
10. What of the following statements concerning temporal lobe surgery are true?
  - Temporal lobectomy is associated with rates ranging from 41% to 51% at 6 months and 21%–34% at 1 year‐ of long‐term language impairments, independently from the side of surgery
  - Limiting the posterior resection margin is sufficient to preserve language functions
  - Lesion–symptom mapping identifies the fusiform gyrus and posterior dominant inferior temporal lobe (4–6 cm from the temporal tip) as critical for naming and reading
  - Atypical (bilateral or right) dominance often predicts better postoperative fluency in right TLE

*Answers may be found in the* *Supporting information*.

## Supporting information


Data S1.

## Figures and Tables

**FIGURE A1:**
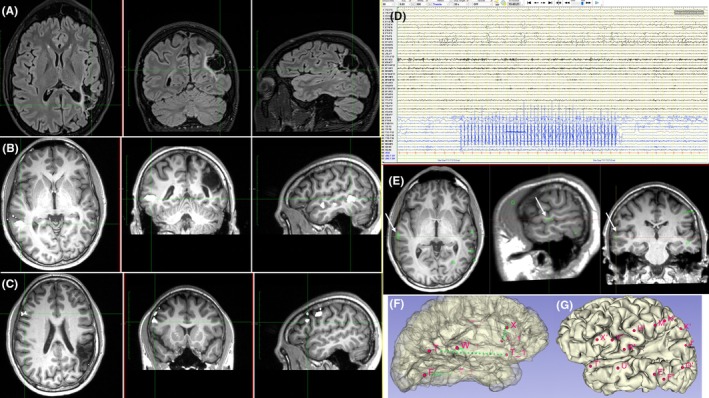
(A) 3D FLAIR T2‐W MRI sequences showing a post‐ischemic encephalomalacia in the left temporo‐parietal region. (B, C) Functional MRI with language‐dedicated tasks (B‐ auditory comprehension, C‐ phonemic and semantic fluency). A slight activation in the right temporal regions (B) and in the right inferior frontal gyrus (C) was observed. Neither cerebellar activation nor activation during the verb generation task was found. (D) Intermediate‐frequency stimulations (frequency 3 Hz, current intensity 5 mA, duration 15 s, pulse width 0.5 msec) on the contacts T11‐12 produced a clear‐cut comprehension deficit. (E) Anatomical localization of the contacts T11‐12 in axial, sagittal and coronal sequences MPRAGE sequences (500.00 μm thickness). (F) 3D Right Stereo‐EEG implantation scheme: Antero‐posterior trajectory of the T electrode. (G) 3D Left Stereo‐EEG left implantation scheme.

**FIGURE A2:**
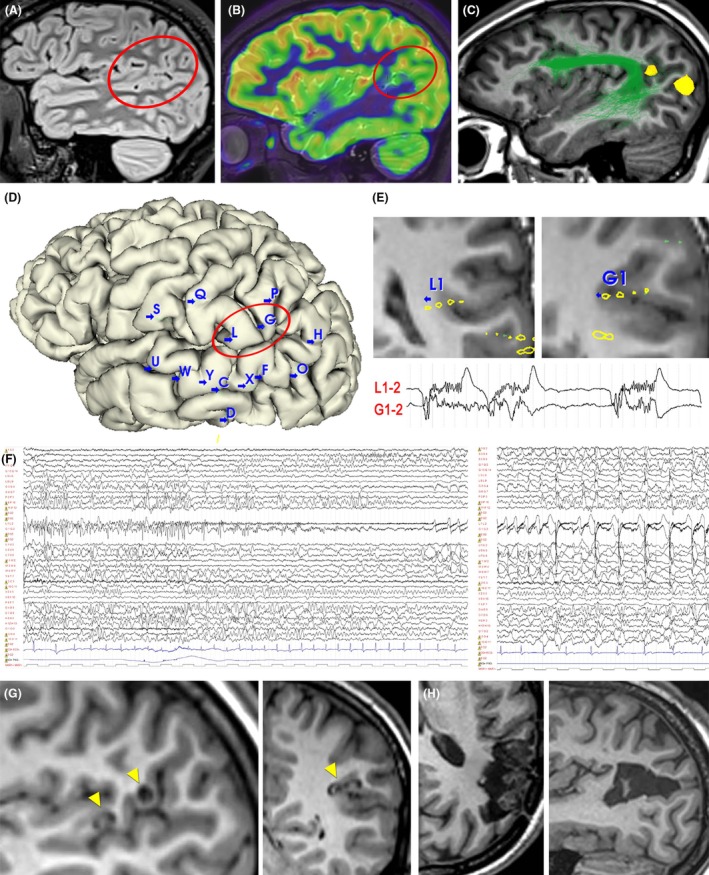
(A) Brain MRI showed focal cortical blurring at the left parieto‐temporal junction. (B) PET imaging confirmed mild hypometabolism in the same region. (C) Functional MRI indicating left‐hemisphere dominance for language production and comprehension. Diffusion tractography showing that the lesion was in proximity to the arcuate fasciculus. (D) Stereo‐EEG electrodes exploring the left temporo‐parietal region. (E) Interictal recording during SEEG. (F) Ictal recordings during SEEG. Seizures originated from a broad network including the parietal cortex, parieto‐occipital junction, fusiform gyrus, and posterior superior temporal gyrus. (G) Brain MRI after thermocoagulation of intralesional electrodes (L3‐4 and G1‐2, G3‐4) produced a 9‐month seizure‐free interval. (H) Postoperative MRI demonstrating complete resection of the dysplasia.

## Data Availability

The data that support the findings of this study are available on request from the corresponding author. The data are not publicly available due to privacy or ethical restrictions.

## APPENDIX A

**Clinical Case 1**

A 24‐year‐old left‐handed man with a drug‐resistant epilepsy was referred to the “Claudio Munari” Epilepsy Surgery Center. Family and personal history were unremarkable. Epilepsy onset occurred at age 12 with onset of focal to bilateral tonic–clonic seizures. Interictal EEG showed slow waves and spikes in left perisylvian regions. The MRI demonstrated post‐ischemic encephalomalacia in the left temporo‐parietal region. Anti‐seizure medications failed to control seizures. He experienced weekly seizures: tongue tingling, bitter taste, and clonic jerking of the right face. He could warn verbally at the onset but was unable to speak during the seizure. He remained dysarthric for a few minutes after seizure. Neuropsychological testing revealed mild deficits in verbal and visuospatial memory. Functional MRI for language did not provide a definitive lateralization/localization of language areas (Las) but suggested right‐hemisphere language dominance.

Stereo‐EEG implantation was planned with 13 intracerebral electrodes to explore the left temporal, insulo‐opercular, and parietal regions, and 4 electrodes exploring the area supposed to be the right LAs. To explore the right posterior LA, one electrode was implanted with an antero‐posterior trajectory along the first temporal gyrus. During Stereo‐EEG recording, high‐frequency stimulations (HFS) and intermediate‐frequency stimulations (IFS) were performed for language mapping, identifying basal and posterior LAs in the right hemisphere.^14^, ^15^ The anterior LA could not be mapped through stimulation of both right/left inferior frontal gyri; moreover, ictal discharges involving the left insulo‐opercular regions (including X') did not induce expressive language disturbances. Based on Stereo‐EEG data, RF‐thermocoagulations were performed in the left insulo‐opercular region without significant deficit but short‐lasting seizure freedom (one month). Therefore, the patient will undergo a left insulo‐opercular cortectomy.

This case highlights the relevance of implanting electrodes contralaterally to the presumed epileptogenic zone, for mapping LAs with IFS, using specific trajectories to provide the necessary clinical data (Figure A1).

**Clinical case 2**

A 15‐year‐old right‐handed girl with normal psychomotor development presented with medically refractory focal epilepsy beginning at age 7 years. Her seizures began with a subjective sensation of dizziness, followed by speech arrest, asymmetric hypertonia, leftward head deviation, smiling, eyelid blinking, and conjugate eye deviation to the left. Neurocognitive assessment was normal.

Brain MRI showed focal cortical blurring at the left parieto‐temporal junction. (B) PET imaging confirmed mild hypometabolism in the same region.

Functional MRI indicated left‐hemisphere dominance for language production and comprehension. Diffusion tractography showed that the lesion was in proximity to the arcuate fasciculus.

A stereo‐EEG electrode was implanted to sample the left temporo‐parietal region. Interictal recordings showed persistent epileptiform discharges (polyspikes, brushes, and suppression) over the internal contacts of electrodes exploring the fundus and upper aspect of the dysplastic sulcus (L1–2, G1–2). Seizures originated from these internal contacts, with ictal activity propagating through a broader network including the parietal cortex, parieto‐occipital junction, fusiform gyrus, and posterior superior temporal gyrus. Clinically, seizures were associated with language impairment, affecting comprehension and especially verbal expression, intermittent elevation of the right upper limb, and nearly continuous rhythmic eyelid blinking. Thermocoagulation of electrodes within the dysplastic sulcus (L3‐4 and G1‐2, G3‐4) produced a 9‐month seizure‐free interval. Given this response, awake surgical resection with intraoperative language monitoring was performed. Postoperatively, the patient developed a naming impairment. Histopathology confirmed focal cortical dysplasia type 2A. At 6‐month follow‐up, MRI demonstrated complete resection of the dysplasia. The patient remained seizure‐free, and her language function had nearly fully recovered, with only mild residual word retrieval difficulty (Figure A2).
