# Supplementary material for: Beyond Broca and Wernicke: Epilepsy surgery in the language areas
Source: Epileptic Disord. 2026 May 30;28(3):581–601. doi: 10.1002/epd2.70282 (PMC13276694; doi:10.1002/epd2.70282)
Supplement: Supplementary file 1 — Data S1. [file EPD2-28-581-s001.docx]

**Answers**

1. Correct answer: c

2. Correct answer: b

3. Correct answer: d

4. Correct answer: a

5. Correct answer: b

6. Correct answer: c

7. Correct answer: c

8. Correct answer: b

9. Correct answer: d

10. Correct answer: c
